# The effect of a 1-year-long intensive Schroth therapy in patients with adolescent idiopathic scoliosis over 45 Cobb degrees who refused surgery in an outpatient clinic in Hungary, a case series

**DOI:** 10.1186/1748-7161-9-S1-O70

**Published:** 2014-12-04

**Authors:** Judit Orban, Krisztina Horvat

**Affiliations:** 1 Scolinea Scoliosis Clinic, Budapest, Hungary

## Background

In curves more than 45 Cobb degrees fusion is considered as basically the only possible treatment although studies about intensive Schroth intervention show that it may be effective in slowing curve progression of patients with Adolescent Idiophatic Scoliosis (AIS).

## Aim

The main purpose of this study was to evaluate the effect of an intensive, 1-year-long and individualized Schroth intervention on curve progression in patients with AIS more than 45 Cobb degrees focused on the efficiency in terms of Cobb angle, axial trunk rotation (ATR) and objective body image.

## Design

Prospective case series design

## Methods

In this case series we included 7 female patients with AIS at the age of 13 with primary thoracic curve, wearing TLSO brace 23 hours/day, Risser: 2-3. The mean Cobb angle at the start of treatment was 47.3° ±2.4°, while the mean ATR with Scoliometer was 14.3° ±3.4°.

Patients received Schroth intervention for 12 months in an outpatient clinic. Schroth therapy was individual and personalized, 45 minutes weekly for each patient, combined with minimum 60 minutes of daily home Schroth exercises 5 times per week. Outcomes were recorded at baseline, 6 and 12 months. Patients are currently in treatment.

## Results

The Cobb angle improved 9.8° in 4 patients, worsened 7° in 1 patient, remained the same 2.5°, in 2 patients. The ATR measurements improved 5.3° in 4 patients, remained the same in 3 patient. Objective body images gave a positive feedback to all patients.

## Conclusion

In conclusion the results of this study confirm the effectiveness of an intensive 1-year-long, individual Schroth intervention for patients with AIS in curves more than 45°. At high risk of progression personalized Schroth intervention appears to be more effective in reducing the progression of scoliosis with additional bracing.

## References

[B1] Negrini S Negrini F Fusco C Zaina F Idiopathic scoliosis patients with curves more than 45 Cobb degrees refusing surgery can be effectively treated through bracing with curve improvements Spine J 2011 11 5 369 80 doi: 10.1016/j.spinee.2010.12.001. Epub 2011 Feb 2 10.1016/j.spinee.2010.12.001 21292562

[B2] Negrini S Zaina F Romano M Negrini A Parzini S Specific exercises reduce brace prescription in adolescent Idiopathic scoliosis: a prospective controlled cohort study With worst-case analysis J Rehabil Med 2008 40 6 451 5 doi: 10.2340/16501977-0195 10.2340/16501977-0195 18509560

[B3] Weiß HR Rigo M Befundgerechte Physiotherapie bei der Skoliose Pflaum Vlg GmbH 2006

